# Giant Chiari’s Network in Healthy Adults

**DOI:** 10.7759/cureus.75254

**Published:** 2024-12-07

**Authors:** Yuko Harada, Atsuo Mori

**Affiliations:** 1 Cardiology, Shonan Atsugi Hospital, Atsugi, JPN; 2 Internal Medicine, Harada Naika Clinic, Kawasaki, JPN; 3 Cardiovascular Surgery, Kawasaki Municipal Hospital, Kawasaki, JPN

**Keywords:** chiari's network, congenital heart disease, echocardiography, eustachian valve, risk factor of stroke

## Abstract

A 40-year-old male visited our clinic for cardiac evaluation. He had palpitations for several years, but the reason was unknown. Transthoracic echocardiography revealed a hyperechoic ribbon-shaped structure that moved vigorously in the right atrium. A contrast CT scan was not able to detect any abnormal structure in the right atrium. Giant Chiari’s network was suspected. Potential risks or implications of Chiari’s network are thrombus formation, embolism, and arrhythmia. If atrial fibrillation (AF) is documented, anticoagulation therapy should be initiated. If an intracardiac thrombus is detected, anticoagulation therapy and/or surgical removal should be considered. Given the absence of atrial fibrillation or thrombus, the necessity of any intervention or anticoagulation remained controversial. Since the Holter monitor did not detect atrial fibrillation, the patient is currently under vigilant observation without medication. The optimal management of asymptomatic Chiari’s network should be established.

## Introduction

Chiari's network is a congenital remnant of the right valve of the sinus venosus. It has been found in 1.3% to 4% of autopsy studies and 2% of patients undergoing transesophageal echocardiography [1]. It is a reticulated network of fibers originating from the Eustachian valve connecting to different parts of the right atrium. The formation of Chiari's network results from incomplete reabsorption of the right valve of the sinus venosus [2]. It is associated with patent foramen ovale (PFO) in 84% of cases [1]. Since 1897, when the anatomist Chiari found a structure in the right atrium connected to the Eustachian valve, clinicians have been obliged to perform a broad differential diagnosis, including the presence of a tumor, vegetation on the tricuspid valve, and an atrial thrombus [3].

As Schneider et al. wrote in 1995, Chiari’s network had been believed to be “generally not of clinical importance,” even though the network may be the site of thrombosis or may cause entrapment of catheters or pacemaker leads [1,4,5]. This belief may still hold true today, as the majority of Chiari's network discovered incidentally during echocardiography is of small size. If it is large, it can be observed with transthoracic echocardiography, but the giant Chiari network is rarely reported. The incidence of giant Chiari’s network is unknown, and its clinical importance is also not known. The risk of stroke will be higher if atrial fibrillation (AF) coexists.

We encountered a case of giant Chiari’s network in a healthy adult showing no sign of thrombosis. PFO was not observed. The other hospital's transthoracic echocardiography showed normal results the previous year. Why a giant Chiari's network appeared in just one year remains a mystery. Intracardiac thrombus and tumor were denied by contrast CT scan. The optimal management for non-symptomatic Chiari's network remains unestablished. Therefore, the necessity of any intervention or treatment for this healthy adult remains controversial.

## Case presentation

A 40-year-old male was referred to our hospital for intermittent palpitation during the previous five years. He underwent a treadmill stress test and a Holter monitor evaluation five years ago and the previous year, both of which were normal. Transthoracic echocardiogram revealed normal in the previous year. Therefore, his palpitation is not considered to be related to cardiac etiology, and he was released for physical occupation. However, his palpitation continued for five years. As he turned 40, he was referred to our hospital for further evaluation for continuous palpitation. He is otherwise healthy with no past medical history.

Physical examination was unremarkable. EKG revealed sinus bradycardia of 51 bpm. Chest X-ray was normal. Laboratory (blood) evaluation, including an assessment of thyroid function to rule out arrhythmia causes, was within normal limits.

**Figure 1:**
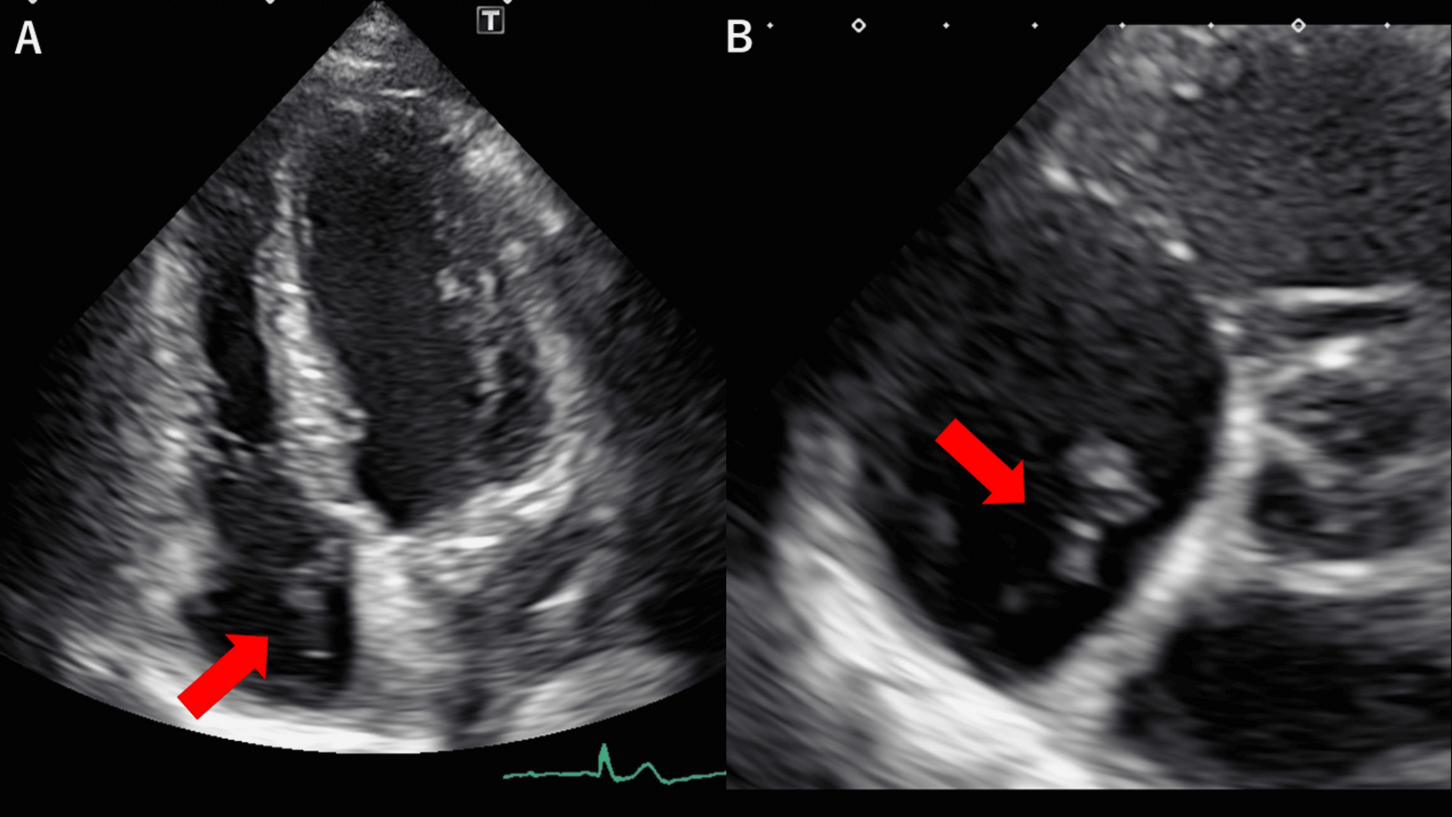
Giant Chiari's network in the right atrium Apex 4 chamber view (A) and short axis view (B) of transthoracic echocardiography. A ribbon-shaped structure is shown in the right atrium (red arrow).

Transthoracic echocardiography revealed a hyperechoic ribbon-shaped structure that was dancing in the right atrium. Figure 1A and Video 1 display apex 4-chamber views. The right atrium displays a ribbon-shaped structure in motion.

**Video 1:** Apex 4 chamber view of transthoracic echocardiography A ribbon-shaped hyperechoic structure is shown dancing in the right atrium. This is the video of Figure 1.

Figure 1B and Video 2 display short-axis views. The right atrium displays a ribbon-shaped structure in motion.

**Video 2:** Short axis view of transthoracic echocardiography A ribbon-shaped hyperechoic structure is revealed dancing in the right atrium, just beneath the tricuspid valve.

**Figure 2:**
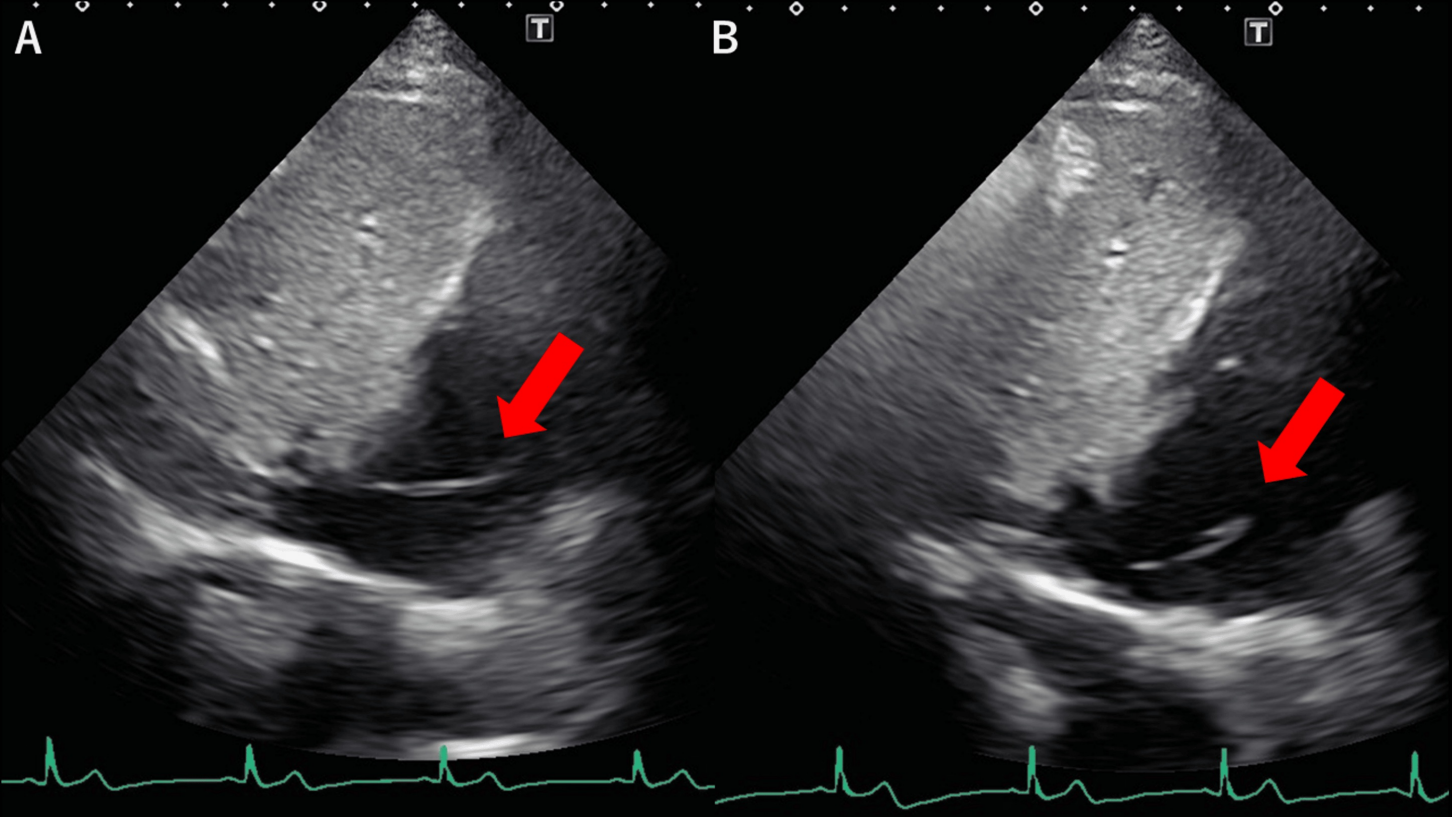
Eustachian valve in the right atrium Transthoracic echocardiography showing IVC. A string-shaped hyperechoic structure (red arrow) is shown in the right atrium near the opening of the IVC. This structure moves up (A) and down (B). It is less mobile than the ribbon-shape structure in Figure 1.

Figure 2 shows the IVC and right atrium. Another string-shaped hyperechoic lesion is observed near the IVC.

PFO was not observed with transthoracic echocardiography. Transesophageal echocardiography was not available in our hospital. A contrast CT scan did not reveal any abnormalities in the right atrium. Therefore, atrial thrombus and tumors were not likely. The ribbon-shaped, rapid, motile structures in Figure 1, Video 1, and Video 2 were suspected to be Chiari’s network. The string-shaped, less-mobile structure in Figure 2 was suspected to be the Eustachian valve.

Holter monitor recording was performed. The result was within normal limits. AF was not detected. As Chiari’s network is innate, it should not be the cause of his palpitation that started five years ago. Even though Chiari's network is dancing in the right atrium, it is not striking the cardiac walls. The cause of his palpitation is still unknown; however, it was considered not to be related to arrhythmia, and actually, tachycardia has not been documented yet. The patient is currently under watchful observation without medication.

## Discussion

The ribbon-like structure in the right atrium was clearly observed with echocardiography but not with a contrast CT scan. Chiari’s network is rapidly moving; therefore, it cannot be detected by a CT scan. The Eustachian valve is difficult to detect with a CT scan due to its very thin structure. The CT scan revealed no thrombus, PFO, or AF, and the patient's CHADS2 score was 0, indicating no need for anticoagulation.

Chiari's network is often clinically insignificant. However, it has been reported to be involved in the pathogenesis of thromboembolic disease, endocarditis, arrhythmias, and entrapment of catheters upon percutaneous intervention [2]. Schneider et al. reported that Chiari's network was present in 29 of 1,436 patients (prevalence 2%) [1]. A frequently associated finding was a PFO in 24 (83%) of the 29 patients with Chiari's network versus 44 (28%) of 160 control patients (p < 0.001) [1]. Therefore, they stated that Chiari's network may favor the persistence of a PFO and the formation of an atrial septal aneurysm and facilitate paradoxical embolism by maintaining an embryonic right atrial flow pattern into adult life, directing the blood from the inferior vena cava preferentially toward the interatrial septum [1]. PFO was not revealed with transthoracic echocardiography in the present case; however, the patient should undergo a microbubble test with transesophageal echocardiography to detect PFO in the near future.

The most recent review of Chiari’s network revealed that the prevalence of the Eustachian valve/Chiari’s network in stroke-related PFO patients was 50% among 883 patients with a mean age of 44.6 years. Patients with a history of stroke had a higher prevalence of the Eustachian valve/Chiari’s network compared with controls, odds ratio = 2.45 [6]. The same review concludes that there is a scarcity of research emphasizing their role in clinical decision-making, especially PFO closure and antithrombotic therapy [6]. In our case presentation, our patient was 40 years old with an Eustachian valve and Chiari’s network but no sign of a stroke. If he has a PFO, he could be at substantial risk for a stroke in the future.

Another review revealed that Chiari’s network and other right atrium remnants may cause entrapment of various devices or catheters during percutaneous cardiac procedures requiring right heart access [7]. Among 41 cases of entrapment, 20% required cardiovascular surgery [7]. Catheter ablation and pacemaker implantation may be dangerous for patients with giant Chiari’s network. Central vein catheter insertion should also be carefully performed in order to avoid proceeding into the right atrium.

Two cases of asymptomatic Chiari’s network coincidentally detected have been reported [8,9]. Both cases did not receive any treatment. One of them started anticoagulation with rivaroxaban, which was discontinued since it did not result in any changes in the echography [9]. However, optimal management for asymptomatic Chiari’s network has not been established yet.

There are some case reports of giant Chiari’s network published in the past [10-12]. A 78-year-old man who had a history of paradoxical embolism underwent surgery due to a giant Chiari's network, which included PFO, severe mitral regurgitation, and severe trigeminal regurgitation [10]. Another case report is a giant Chiari network that mimics an intracardiac tumor [11]. A case of an 80-year-old woman presented with fulminant bilateral pulmonary thromboembolism and a serpentine-like huge mass in the right atrium [12]. The ratio or incidence of a large Chiari network is not known. However, these case reports suggest that the giant Chiari network may cause embolism later in the patients’ lives.

In our case presentation, the patient presented with palpitations for several years. The cause of his palpitation is still a mystery; however, his palpitation is still going on. He wonders if he can safely continue his occupation with physical activities. Even though the Holter monitor did not detect AF, our patient could have had paroxysmal AF, which the Holter may have failed to record. It is therefore recommended to use a self-monitoring watch device or to repeat a Holter monitor evaluation every three to six months. If AF is detected, anticoagulation should be initiated.

Cardiac echography should also be repeated every 6-12 months to detect thrombus in the right atrium. If a PFO is not detected with transthoracic echocardiography, transesophageal echocardiography and microbubble tests are recommended. However, transesophageal echocardiography is somewhat invasive; therefore, it is not urgently required and not necessary to be repeated for such patients with a lack of symptoms. It is also a mystery why such a large Chiari network was not detected the previous year by echocardiography. It could be due to technical problems or mechanical problems. Otherwise, Chiari network became apparent with thickened or calcified fibers. Contrast CT scan is not useful to detect ribbon-shaped, rapidly moving structures in the heart, just as the cardiac valves are not detected due to their fast movement.

It is also important to educate the patient about an optimal lifestyle, including diet and exercise, in order to prevent hypertension or diabetes.

## Conclusions

Giant Chiari’s network was incidentally found during the evaluation for palpitation. The arrhythmia was not detected, and the cause of his palpitation was not revealed. The Chiari’s network was large-sized and remarkable; however, it was not detected in the previous year. Optimal management for asymptomatic Chiari’s network was suggested as follows: repeating Holter monitor every three to six months to detect AF, repeating echocardiography every 6-12 months, microbubble tests to detect PFO, avoiding percutaneous cardiac procedures that require right heart access (if alternative access routes are available or if the procedure involves potential entrapment risks), and improving lifestyle habits such as diet and exercise. If AF is documented, anticoagulation should be initiated to prevent a stroke. The establishment of optimal management of asymptomatic Chiari’s network is anticipated. Further research is anticipated, such as studies on the natural history of Chiari’s network or its clinical outcomes. Updated guidelines or consensus statements on managing incidental findings of Chiari’s network are also needed.
